# The Role of TRPC6 in Renal Ischemia/Reperfusion and Cellular Hypoxia/Reoxygenation Injuries

**DOI:** 10.3389/fmolb.2021.698975

**Published:** 2021-07-08

**Authors:** Xin Hou, Mengjun Huang, Xixi Zeng, Yanhong Zhang, Anbang Sun, Qifang Wu, Lin Zhu, Hu Zhao, Yanhong Liao

**Affiliations:** ^1^ Department of Anatomy, Medical College, Affiliated Hospital, Hebei University of Engineering, Handan, China; ^2^ Department of Anatomy, Tongji Medical College, Huazhong University of Science and Technology, Wuhan, China

**Keywords:** AKI, renal ischemia/reperfusion injury, TRPC6, apoptosis, autophagy

## Abstract

Renal ischemia/reperfusion (I/R), a major cause of acute kidney injury (AKI), is a serious clinical event in patients during post-renal transplantation. I/R is associated with renal dysfunction and tubular apoptosis, and calcium (Ca^2+^) overload has been reported to be a crucial factor on tubular apoptosis in I/R injury (IRI). The canonical transient receptor potential channel 6 (TRPC6), a type of non-selective Ca^2+^ channel, is involved in many renal diseases. Our earlier study identified that TRPC6-mediated Ca^2+^ influx plays a novel role in suppressing cytoprotective autophagy triggered by oxidative stress in primary tubular epithelial cells (TECs). This study explored the potential beneficial impact of TRPC6 knockout (TRPC6^−/−^) and the relevant cellular mechanisms against I/R-induced AKI in mice. Measuring changes of renal function, apoptotic index, and autophagy in mouse kidneys that suffered 24 h reperfusion after 40 min ischemia and working *in vitro* with TECs that suffered 24 h reoxygenation after 24 h hypoxia, we found that 1) IRI tissues had increased TRPC6 expression and TRPC6 knockout significantly ameliorated renal damage induced by IRI; 2) TRPC6 knockout enhanced the level of autophagy and alleviated the depolarization of mitochondrial membrane potential (ψm, MMP) and apoptotic changes upon IRI; and 3) IRI tissues had increased p-AKT and p-ERK1/2 expressions, while TRPC6 knockout could markedly reduce the phosphorylation of AKT and ERK1/2. These discoveries suggest that, by reducing Ca^2+^ overload, the underlying protective mechanism of TRPC6^−/−^ may be involved in down-regulation of PI3K/AKT and ERK signaling, which is likely to provide a new avenue for future AKI therapies.

## Introduction

Renal ischemia/reperfusion injury (IRI) is a serious syndrome characterized by impaired renal function and associated with high morbidity of accelerating chronic kidney disease (CKD) that may progress to end-stage renal disease (ESRD) and increased mortality as a consequence of multi-organ failure (Polichnowski et al., 2020). An increase of serum creatinine (Scr) ≥1.5-fold from baseline renal function within the prior 7 days is defined as acute kidney injury (AKI) according to the KDIGO guidelines (Khwaja, 2012). The process of renal ischemia/reperfusion (I/R) is a succession of cellular events that can be divided into two distinct partial steps known as ischemia and reperfusion. During the reperfusion phase, temporarily interrupted blood flow was restored, leading to the imbalanced production of reactive oxygen species (ROS) and significant down-regulation of the antioxidant enzyme system, eventually accompanying secondary deterioration of tissue damage (Wu et al., 2018). Renal tubular epithelial cells (TECs) are involved as sensors, effectors, and victims of IRI because of the rich energy metabolisms of the kidney and the role of reabsorption and concentration after glomerular filtration. With the increasing duration of ischemia, the kidney will have more loss of TECs into the lumen, more serious disruption of renal function, and increased necrosis and apoptosis (Molitoris, 2014). Abundant evidence has shown that oxidative stress, calcium (Ca^2+^) overload, and mitochondrial dysfunction are key contributors to cell damage and apoptosis during IRI (Zhong et al., 2018; Kamarauskaite et al., 2020). Thus, it is essential that minimizing the Ca^2+^ accumulation–induced apoptosis of TECs will contribute to the recovery of TEC integrity and function when undergoing equal injuries (Sharfuddin and Molitoris, 2011; Poston and Koyner, 2019).

Canonical transient receptor potential channels (TRPCs), characterized by non-selective Ca^2+^-permeable cation channels, are members of the TRP superfamily. It is widely recognized that store-operated calcium entry (SOCE) is involved in Ca^2+^ regulation and TRPCs participate in SOCE (Liao et al., 2008; Lewis, 2020). The TRPC subfamily contains seven members in mammals (TRPC1–TRPC7), among which TRPC6 has been reported to distribute in the plasma membrane of kidney cells, including podocytes, glomerular mesangial cells, and TECs (Reiser et al., 2005). Prior studies have revealed that TRPC6 is associated with pathophysiology of podocyte apoptosis induced by I/R. Moreover, our previous data have suggested that TRPC6 is involved in renal proximal tubular cell apoptosis upon H_2_O_2_ (Hou et al., 2018). Additionally, our previous studies identified that TRPC3/6/7 knockout mice ameliorated myocardial or brain injury *via* protection of myocardial cells or astrocytes from apoptosis when exposed to IRI (Chen et al., 2017; He et al., 2017). Based on these data, we reconfirmed the detrimental effect of TRPC6 sufficiently on TECs through establishing both the *in vivo* I/R model and the *in vitro* hypoxia/reoxygenation (H/R) model.

Apoptosis, characterized by cell shrinkage, chromatin condensation, nuclear fragmentation, and apoptotic bodies, is a significant event involved in I/R-induced AKI. It has been shown that the genes that participate in apoptotic regulation include caspase and B-cell lymphoma-2 (Bcl-2) family proteins (Green and Llambi, 2015). Calcium, as one of the second messengers, plays a critical role in various physiological processes, including cell contraction, signal transduction, gene regulation, and apoptosis. Numerous publications have shown that changes in Ca^2+^ concentration following I/R can lead to mitochondrial Ca^2+^ accumulation associated with mitochondrial dysfunction and result in cell apoptosis and necrosis (Agarwal et al., 2016; Bhargava and Schnellmann, 2017; Kuznetsov et al., 2019). Exploration of the signaling pathways of I/R-induced cell apoptosis would shed light on the mechanisms of TEC injury.

Autophagy, a highly conserved lysosomal-dependent catabolic process, is mainly responsible for the removal of misfolded proteins and damaged organelles and plays an important role in maintaining cell homeostasis during pathologic stress conditions, such as renal I/R injury (Decuypere et al., 2015). Dong *et al.* have shown that autophagy plays a crucial role in TEC damage induced by renal I/R (Tang et al., 2018). Autophagy can be directly activated by oxidative stress and ROS during renal I/R (Havasi and Dong, 2016). As a highly conserved lysosomal-dependent degradation pathway, autophagy enables energy regeneration and cellular component reuse by degrading damaged or toxic proteins, lipids, and organelles. Under the influence of renal I/R, TECs with high metabolic rate are the primarily targeted cells to be damaged. Therefore, TECs must be highly dependent on autophagy to cope with external stressors to maintain their stability. Autophagy itself is a highly dynamic, multi-stage process. The basic processes of autophagy include autophagy initiation, membrane extension, formation of autophagosomes, fusion of autophagosomes with lysosomes, and degradation of substrates. Therefore, to estimate the change of autophagy, it is necessary to evaluate the change of the whole process, which is defined as “autophagic flux.” Recently, we have shown that the increase of calcium influx mediated by TRPC6 can inhibit autophagic flux in TECs upon oxidative stress (Hou et al., 2018). However, the interaction and molecular mechanism between TRPC6 and autophagy in renal IRI have not been reported. The main hypothesis of our study was that calcium influx mediated by TRPC6 inhibits protective autophagy and promotes apoptosis of TECs upon IRI.

Hypoxia inducible factor-1 (HIF-1) is a key transcription factor mediating adaptive responses to hypoxia during I/R. In response to hypoxia in the kidney, HIF-1α is expressed predominantly in TECs and works as a master regulator of hypoxic stress (Choudhry and Harris, 2018). TRPC6 is known to regulate the metabolism to affect HIF-1α stability and consequent glucose metabolism in certain cancer cell types such as glioma cells under hypoxic conditions (Li et al., 2015). In contrast, it is unknown that how does TRPC6 influence the levels of HIF-1α and its downstream effector protein Aurora kinase A (AurA) in TECs upon I/R.

In order to distinctly comprehend the relationship between I/R-induced AKI and TRPC6, the current study developed *in vivo* and *in vitro* models to focus on whether TRPC6 participates in I/R-induced TEC apoptosis and to probe the underlying mechanism. Our results demonstrated that TRPC6-mediated Ca^2+^ entry inhibits cytoprotective autophagy and promotes TEC apoptosis causing the deterioration of renal function upon IRI. Moreover, a molecular mechanism analysis of the *in vitro* H/R model revealed that eliminating TRPC6-mediated Ca^2+^ entry could suppress the activity of PI3K/AKT and ERK pathways, thus facilitating TEC survival.

## Materials and Methods

### Chemicals and Reagents

Sources of antibodies and reagents are as follows: fetal bovine serum (FBS) (Invitrogen), DMEM/F12 (Invitrogen), anti-cleaved caspase 3 (Cat#: 9661, Cell Signaling Technology), anti-Bcl-2 (Cat#: 12789-1-AP, Proteintech), anti-Bcl-2–associated X (BAX) (Cat#: 50599-2, Proteintech), p-AKT (ser473) (Cat#: 4060P, Cell Signaling Technology), AKT (Cat#: 9272, Cell Signaling Technology), p-ERK1/2 (Cat#: 4370, Cell Signaling Technology), ERK1/2 (Cat#: 4695, Cell Signaling Technology), TRPC6 (Cat#: ACC-017, Alomone), polycystin 2 (Cat#: 19126-1-AP, Proteintech), AURKA (Cat#: 66757-1-lg, Proteintech), HIF-1α (Cat#: 79233, Cell Signaling Technology), p62 (Cat#: 88588, Cell Signaling Technology), β-actin (Cat#: TA-09, ZhongShan Biotechnology), HRP-conjugated anti-rabbit IgG (Cat#: 110777, KeRui Biotechnology), HRP-conjugated anti-mouse IgG (Cat#: 117228, KeRui Biotechnology), U0126 (Cat#: HY-12-31, MedChemExpress), MK2206 (Cat#: HY-10358, MedChemExpress), thapsigargin (Tg) (Cat#: T7459, Invitrogen), JC-1 dye (Bio-Swamp), Creatinine (Cr) Assay Kit (sarcosine oxidase) (Cat#: C011-2, Nanjing Jiancheng), Urea Assay Kit (urease) (Cat#: C013-2, Nanjing Jiancheng), and OCT compound (4583, Sakura). DMEM/F12 and FBS were purchased from Invitrogen (Chicago, California, United States). The whole sagittal section of the kidney was scanned by Biossci Biotechnology Company (Wuhan, Hubei, China).

### Mice Models

TRPC6^−/−^ mice on a 129SvEv background were reconstituted at the Comparative Medicine Branch (CMB) of the National Institute of Environmental Health Sciences (NIEHS), North Carolina, United States WT mice, which served as controls for the knockout mice, were also obtained from the NIEHS. Mice were permitted *ad libitum* access to food and water. Mice were kept on a 12-h light/12-h dark cycle in a temperature-controlled room. The study was conducted on adult male 8- to 10-week-old TRPC6^−/−^ mice weighing 20–25 g and their age-matched WT 129SvEv controls. The animal studies were reviewed and approved by the Ethics Committee of Tongji Medical College (Huazhong University of Science and Technology).

### *In Vivo* Ischemia/Reperfusion: Mouse Model of Renal I/R

Sixty WT (129SvEv) and sixty TRPC6^−/−^ mice were randomly and equally divided into the following groups: 1) Group I: WT-sham (*n* = 30), 2) Group II: WT-I/R (*n* = 30), 3) Group III: TRPC6^−/−^-sham (*n* = 30), and 4) Group IV: TRPC6^−/−^-I/R (*n* = 30). Mice were fasted for 8–10 h before surgery, then anesthetized with 3% chloral hydrate (10 ml/kg), and fixed on the heating plate in the back upward position. Along the left and right sides of the spine, 1.5 cm longitudinal incisions were made. The kidneys were gently squeezed out upward, and the renal pedicles were carefully separated. The bilateral renal pedicles were then closed with non-invasive microarterial clamps for 40 min. After 40 min of ischemia, reperfusion was performed for 24 h. The renal pedicles of the sham groups were separated but not clamped, and the other operations were the same as in the I/R model groups.

### Renal Function Measurement

Following 24 h of reperfusion, blood samples were taken immediately after the eyeballs of the experimental mice were removed. The serum was obtained by centrifugation at 4,000 × g for 5 min. The levels of serum creatinine (Scr) and blood urea nitrogen (BUN) were determined by a biochemical auto-analyzer (Sysmex CHEMIX-180) using the commercial diagnostic kits.

### Immunofluorescence Staining

Tissue sections or cell glass slides were washed three times (10 min each time) with 0.01 M PBS and fixed with 4% paraformaldehyde for 15 min. Tissues or cells were permeabilized with 0.1% Triton X-100 for 10–20 min. After being washed with PBS in the same way, they were incubated with active caspase 3 and anti-TRPC6 for 2 h and then incubated with goat anti-rabbit FITC for another 2 h in the dark. The nuclei were stained with 4′,6-diamidino-2-phenylindole (DAPI) for 5 min followed by PBS in the same way. Next, an anti-fluorescence attenuation sealant was used to seal the tissue sections or cell glass slides, and a laser scanning confocal microscope was used to take pictures.

### Isolation and Primary Culture of Renal Tubular Epithelial Cells

The cell culture method is borrowed from our previous article (Hou et al., 2018). Mice aged 8–10 weeks were sacrificed for cervical dislocation and disinfected with 75% alcohol. The kidneys were quickly removed and transferred to cold D-Hanks’ Balanced Salt Solution with 1% penicillin–streptomycin. After stripping the outer membrane of the kidneys, the renal cortex was carefully separated from the medulla and then minced into small pieces and digested with 2.5 ml D-Hanks solution containing 0.1% collagenase II for 10 min, four times at 37°C. All supernatant was passed through two nylon screens with different diameters of 180 and 75 μm. The cells left at 75 μm were resuspended with D-Hanks solution and centrifuged at 800 rpm for 5 min. After centrifugation, the cells were resuspended with culture medium DMEM/F12 containing 10% fetal bovine serum, seeded in required size culture dishes or glass slides, and incubated in a stationary state for 72 h in 95% O_2_–5% CO_2_ at 37°C. Then, the medium was replaced according to the growth of the cells, and the corresponding treatment was done.

### Hypoxia/Reoxygenation Experiment

After 4 days, TECs formed a single layer of confluence under normal atmosphere (containing 5% CO_2_, 21% O_2_, and 74% N_2_) in a DMEM/F12 mediumcontaining 10% fetal bovine serum. TECs were subjected to hypoxia/reoxygenation (H/R) experimentation in order to simulate the I/R model *in vitro*. Then, the medium above was replaced by Tyrode’s solution without glucose (0.37 g KCl, 2.88 g NaCl, 0.06 g CaCl_2_, 0.15 g MgSO_4_·7H_2_O, 2.81 ml sodium lactate, 2.38 g HEPES, and 500 ml ddW, pH 6.8), and the cells were incubated in anaerobic conditions (containing 5% CO_2_ and 95% N_2_) for 24 h. The cells were transferred to normal culture conditions including medium and air during reoxygenation for 24 h. During reoxygenation, the inhibitor group cells were treated with selective blockers of ERK (U0126, 25 μM) and AKT (MK2206, 5 μM).

### Histology and Histopathology

The kidneys were removed at a predetermined time, then were fixed in 4% paraformaldehyde, dehydrated by 10, 20, and 30% sucrose step by step, embedded in the OCT compound, and serially sectioned at 5 μm thickness. The tissue sections were stained with hematoxylin and eosin (H&E) and periodic acid–Schiff (PAS) in accordance with standard experimental procedures. The tissue sections were photographed at a magnification of ×200. Representative renal tubules were selected by careful viewing. All the procedures were conducted by three blinded pathologists.

### Calcium Imaging Test

Calcium imaging was performed as previously described (Hou et al., 2018). Briefly, the cells were loaded with 3 μM Fura2-AM in a DMEM/F12 medium at 1:1 for 50 min at room temperature. Then, the cells were washed three times with an HBSS medium (140 mM NaCl, 5 mM KCl, 10 mM HEPES, 10 mM glucose, and 1 mM MgCl_2_, pH 7.4) with 2 mM Ca^2+^ and incubated at room temperature for another 10 min. The coverslips were mounted onto the platform of an inverted epifluorescence microscope. Cytosolic Ca^2+^ was monitored with an Olympus IX51 inverted fluorescence microscope and SlideBook software, using excitation wavelengths of 340 and 380 nm to detect Fura-2/Fura2-Ca^2+^ fluorescence emissions at 510 nm.

### Mitochondrial Membrane Potential Detection

After TEC H/R injury in the six-well plate, the mitochondrial membrane potential was calculated. 1 ml JC-1 dye working solution was added to 1 ml medium for 20 min at 37°C in the dark. After incubation, the plates were washed with JC-1 buffer twice. Fluorescence was visualized and pictured first at emission 595 nm (red) and then at 529 nm (green) using a fluorescence microscope. The proportion of mitochondrial depolarization was measured by the relative ratio fluorescence intensity of red and green.

### Western Blot Analysis

Lysis buffer (50 mM Tris-HCl (pH 6.8), 150 mM NaCl, 1 mM EDTA, 1% NP-40, and 1 mM PMSF) and RIPA buffer (150 mM NaCl, 50 mM Tris-HCl, 1% Triton X-100, 0.5% sodium deoxycholate, 0.1% SDS, and 1 mM EDTA, pH 7.4) were used to lyse the TECs and the renal cortex, respectively. Cell and tissue lysate concentrations were measured according to the instructions of the BCA kit. The protein sample was mixed with loading buffer and boiled for 5–10 min. After polypropylene gel electrophoresis for the same amount of protein samples, the PVDF membrane was transferred and sealed with 5% skim milk for 1.5 h. The PVDF membranes were incubated with corresponding primary antibodies at 4°C for 8–24 h. After 30 min washing with TBST, the membranes were incubated with appropriate horseradish peroxidase (HRP)-conjugated secondary antibody for 1.5 h at room temperature. After another 30 min washing with TBST, the membranes were developed with ECL solution and measured with the NIH ImageJ software.

### Statistical Analyses

All experiments were performed in triplicate at least three times, and the results were expressed as mean ± SEM (standard error of the mean). The statistical differences between groups were assessed by using one-way analysis of variance (ANOVA) depending on the number of comparisons being made and the data distribution. When *p*-values < 0.05, the differences were considered significant.

## Results

### Expressions of TRPC6 Are Increased in Both I/R and H/R Injuries

**FIGURE 1:**
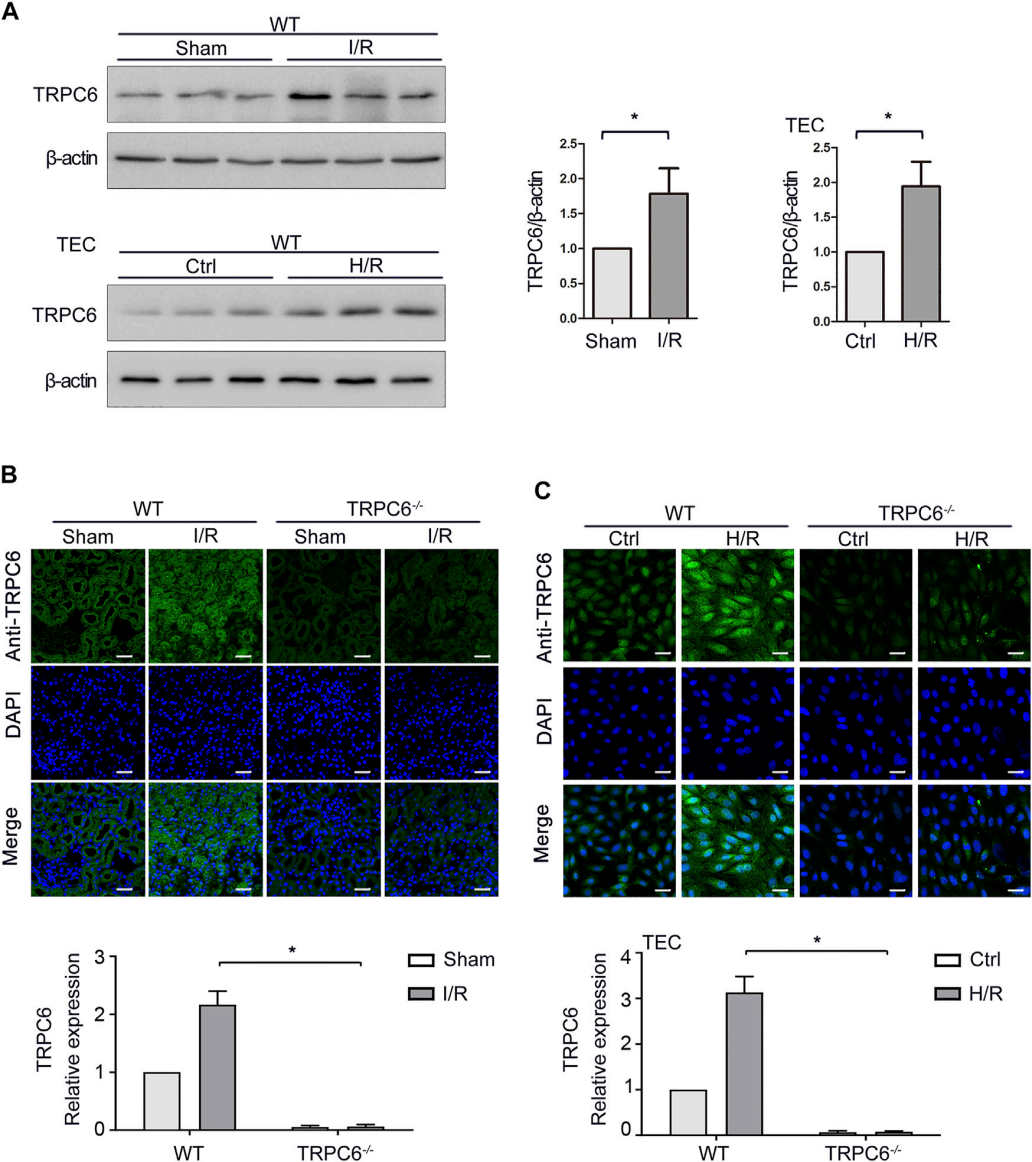
Increased expression of TRPC6 after I/R and H/R injuries. **(A)** Representative western blot images of TRPC6 in renal tissue lysates of WT-sham and WT-I/R and TEC lysates of WT-H/R treatment. Bars represent mean ± SEM, *n* = 3, ^*^ *p* < 0.05. **(B)** Representative confocal microscopy images of TRPC6 expression in kidney sections from WT and TRPC6^−/−^ mice subjected to sham or I/R operations (photographed at a magnification of ×200, scale bar: 50 μm). Bars represent mean ± SEM, *n* = 6, ^*^ *p* < 0.05. **(C)** Representative confocal microscopy images of TRPC6 expression in primary TECs from WT and TRPC6^−/−^ mice upon H/R treatment (photographed at a magnification of ×200, scale bar: 50 μm). Bars represent mean ± SEM, *n* = 6, ^*^ *p* < 0.05.

TRPC6 is generally expressed in renal cells, including renal tubular epithelial cells (TECs). Our previous study illustrated that SOCE exists in TECs and TRPC6 participates in Ca^2+^ release and Ca^2+^ influx in TECs induced by oxidative stress (Hou et al., 2018). On this basis, we further examined the changes of TRPC6 in acute renal injury both *in vivo* and *in vitro*. To assess whether I/R was associated with an increase of TRPC6 in TECs, the expression of TRPC6 in renal tissue lysates of WT-sham and WT-I/R mice was measured by western blot. As shown in Figure 1A, TRPC6 protein levels were elevated in WT-I/R mice compared with the WT-sham group. To evaluate whether I/R-induced increase of TRPC6 also appears in the model of H/R *in vitro*, TECs were subjected to H/R injury to simulate the I/R model. Likewise, changes of TRPC6 proteins were increased after H/R treatment, which was in accordance with that for mice subjected to I/R injury *in vivo* (Figure 1A). In order to provide further evidence of this change, we performed tissue and cellular immunofluorescence detection. Immunofluorescence labeling of tissue sections was studied in WT and TRPC6^*−/−*^ mice. We found that TRPC6 expression was increased in WT mice after I/R operations, which was confirmed to be weak in TRPC6^*−/−*^ mice regardless of operations in comparison with WT mice (Figure 1B). This may be due to the non-specific signal of TRPC6 antibody immunofluorescence. Meanwhile, immunofluorescence analysis of TECs separated from WT and TRPC6^*−/−*^ mice after H/R treatment was measured. As can be seen, TRPC6 expression of WT mice was obviously augmented upon H/R process. However, TRPC6^*−/−*^ mice exhibited weak labeling of TRPC6 upon H/R which was in agreement with the above results (Figure 1C). All data showed that TRPC6 was involved in I/R and H/R processes and that the expression of TRPC6 protein was increased in I/R and H/R injuries.

### TRPC6 Knockout Attenuates Renal Tubular Damage Upon IRI *In Vivo*

**FIGURE 2:**
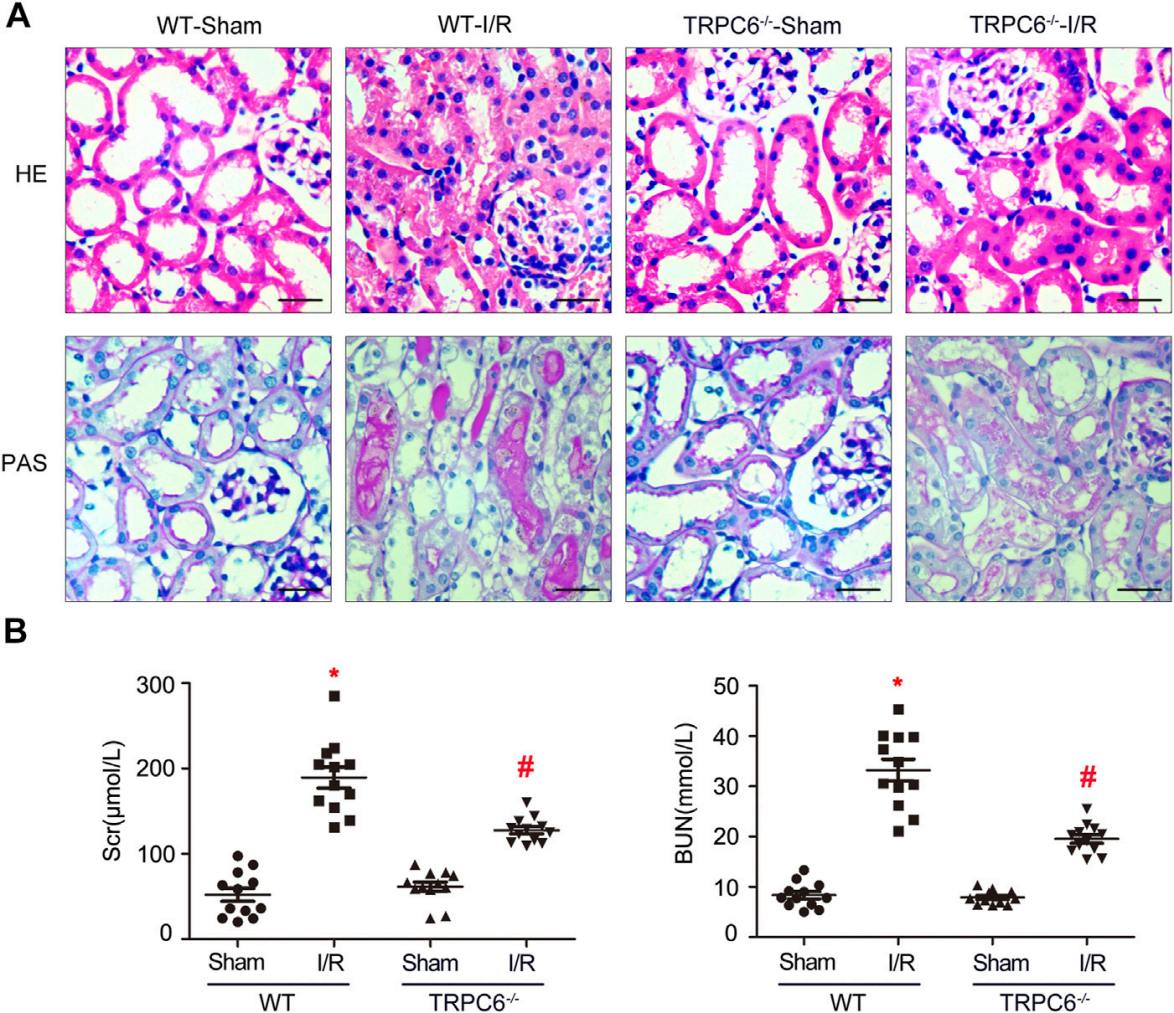
TRPC6 knockout attenuates renal tubular damage upon IRI *in vivo*. **(A)** Representative images of kidney tissue section samples of WT and TRPC6^−/−^ mice stained with H&E and PAS (photographed at a magnification of ×200, scale bar: 50 μm). **(B)** Assessment of BUN and Scr levels. Values are expressed as mean ± SEM (*n* = 12 mice per group); ^*^ *p* < 0.05 *vs.* WT-sham; ^#^ *p* < 0.05 *vs.* WT-I/R, Table 1. Renal function measurement of WT and TRPC6^−/−^ mice after 24 h reperfusion following 24 h ischemia. Values are expressed as mean ± SEM (*n* = 12 mice per group); ^*^ *p* < 0.05 *vs.* WT-sham; ^#^ *p* < 0.05 *vs.* WT-I/R.

In the AKI model induced by IRI, renal tubules resulted with severe changes after kidney injury. It was confirmed that inflammatory responses induced by IRI are dependent on neutrophils and pro-inflammatory cytokines (Zhou et al., 2018). To deeply investigate the effects of TRPC6 during I/R injury, WT and TRPC6^−/−^ mice were subjected to sham or I/R operations, and then, kidney and blood samples were obtained after 24 h of reperfusion. H&E staining and PAS staining were performed on histological sections. From the histological analysis by H&E staining, the appearance of the sections in the WT-I/R group featured with serious morphological alterations, including a large number of TEC swelling, vacuolization and disordered arrangement, impaired brush border shedding into the tubular lumen, and heavy tubular necrosis. As compared to WT-I/R, TRPC6^*−/−*^ ***-***I/R resulted in slight structural changes and had less tubular necrosis (Figure 2A). Strikingly, histological findings of PAS staining were identical to those of H&E staining (Figure 2A). To better clarify the protective role of TRPC6^*−/−*^, we next focused on the comparison of renal function using Scr and BUN tests in the subsequent studies. There were no significant differences in the value of Scr and BUN between WT-sham and TRPC6^*−/−*^ ***-***sham. Particularly, Scr quickly improved to the level of 189.58 ± 42.827 μM and BUN increased to the level of 33.235 ± 7.508 mM in WT-I/R compared with WT-sham (Table 1). Also, the range of Scr and BUN was greater in WT-I/R than TRPC6^*−/−*^ ***-***I/R, namely, TRPC6 has the potential of developing worsened renal dysfunction in I/R circumstance (Figure 2B). All of these findings illustrate that TRPC6^*−/−*^ attenuates renal tubular damage in the model of IRI *in vivo*.

**TABLE 1:** Renal function measurement of WT and TRPC6^−/−^ mice after 24 h reperfusion following 24 h ischemia.

Variable	WT-sham	WT-I/R	TRPC6^−/−^-sham	TRPC6^−/−^-I/R
N	12	12	12	12
SCR (μMP)	51.956 ± 26.57	189.58 ± 42.82*	61.448 ± 18.96	127.58 ± 12.82^#^
BUN (MMP)	8.336 ± 2.49	33.235 ± 7.50*	7.878 ± 1.31	19.542 ± 2.92^#^

Values are expressed as mean ± SEM (*n* = 12 mice per group); *p < 0.05 *vs*. WT-sham; ^#^ *p* < 0.05 *vs*. WT-I/R.

### Expressions of Autophagy- and Apoptosis-Related Proteins Are Increased Upon IRI

**FIGURE 3:**
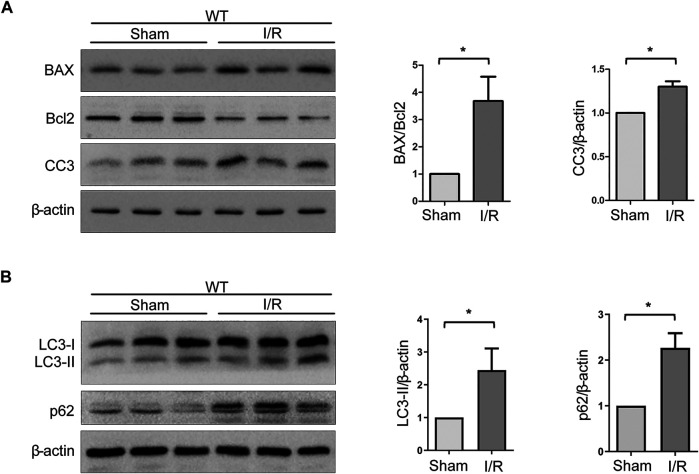
Expressions of autophagy- and apoptosis-related proteins are increased upon IRI. **(A)** Representative western blot images of BAX, Bcl-2, and CC3 expressions in renal tissue lysates of WT mice subjected to sham or I/R operations. Bars represent mean ± SEM, *n* = 3, ^*^ *p* < 0.05. **(B)** Representative western blot images of LC3-II and p62 expressions in renal tissue lysates of WT mice subjected to sham or I/R operations. Bars represent mean ± SEM, *n* = 3, ^*^ *p* < 0.05.

The Bcl-2 family and caspase 3 have been reported to be of vital importance in the regulation of apoptosis. BAX and cleaved caspase 3 (CC3), which are pro-apoptosis proteins, accelerate apoptosis, while Bcl-2 is an anti-apoptosis protein that promotes survival. Western blot analysis of renal tissues has shown that the level of BAX and CC3 significantly increased and Bcl-2 decreased after WT mice suffered from the I/R protocol (Figure 3A). Data analysis of BAX/Bcl-2 ratio, an index of pro-apoptosis, indicated a dramatic rise upon I/R (Figure 3A). In addition, it is known that autophagy plays a crucial role in renal I/R. Our previous experiments have shown that autophagy participates in oxidative stress–induced TEC damage. Here, we go further to investigate the role of autophagy in renal I/R injury *in vivo*. Microtubule-associated protein 1 light-chain 3 (LC3)-II is the most widely monitored autophagy-related protein. During autophagy, LC3-II protein binds to the inner membranes of the autophagosome and is conveyed and degraded in the autolysosome, making it a good marker to rate autophagic flux (Nakatogawa et al., 2009). Ubiquitin-associated protein p62/sequestosome-1 (SQSTM1), an important selective autophagic adaptor protein, is also used to monitor autophagic flux. P62, which binds to the ubiquitinated substrate and LC3, is degraded by proteolytic enzymes during lysosomal degradation. Therefore, elevated p62 levels are generally considered to be a marker of inhibited autophagic flux (Sharifi et al., 2015). In this study, western blot analysis of renal tissues has shown that the levels of LC3-II and p62 were significantly increased upon I/R (Figure 3B). These findings indicated that autophagic flux is impaired in the model of renal IRI.

### TRPC6 Knockout Enhances Autophagy and Mitigates Apoptosis Upon I/R Treatment

**FIGURE 4:**
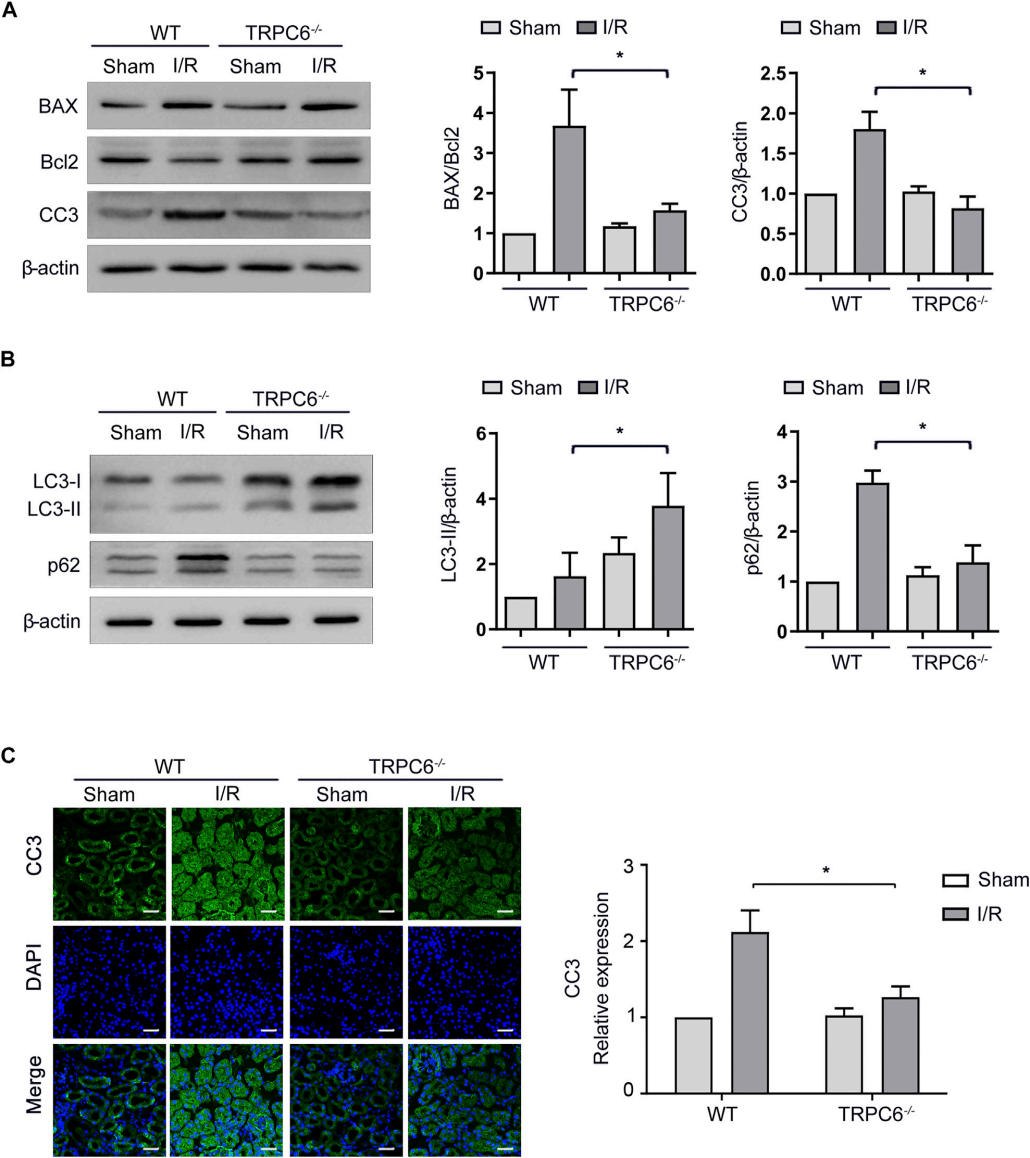
Autophagy is augmented and apoptosis is mitigated in TRPC6^−/−^ mice upon I/R treatment. **(A)** Representative western blot images of BAX, Bcl-2, and CC3 expressions in renal tissue lysates extracted from WT and TRPC6^−/−^ mice subjected to I/R operations. Data are expressed as mean ± SEM, *n* = 3, ^*^ *p* < 0.05. **(B)** Representative western blot images of LC3-II and p62 expressions in renal tissue lysates extracted from WT and TRPC6^−/−^ mice subjected to I/R operations. Data are expressed as mean ± SEM, *n* = 3, ^*^ *p* < 0.05. **(C)** Representative confocal microscopy images of CC3 expression in kidney sections from WT and TRPC6^−/−^ mice subjected to sham or I/R operations (photographed at a magnification of ×200, scale bar: 50 μm). Bars represent mean ± SEM, *n* = 6, ^*^ *p* < 0.05.

Under the condition that the level of apoptosis increases after I/R injury, we further carried out the I/R experiment between WT and TRPC6^*−/−*^ mice. As illustrated in Figure 4A, the enhanced levels of BAX and CC3 from TRPC6^*−/−*^ mice upon I/R protocol were much lower than those in WT mice with the same operations. Different from the reduced level of Bcl-2 in WT-I/R, it had a marked increase in TRPC6^*−/−*^ ***-***I/R. Likewise, compared with that in WT-I/R, the ratio of BAX/Bcl-2 in TRPC6^*−/−*^-I/R was lower (Figure 4A). Furthermore, there was no observed difference in apoptosis between the WT and TRPC6^*−/−*^-sham groups. To explore the function of TRPC6 in I/R-mediated autophagy, western blot analysis of LC3-II and p62 was conducted. The results showed that the level of LC3-II was significantly increased, while p62 remarkably decreased in TRPC6^*−/−*^ mice compared with those in WT mice (Figure 4B), indicating that TRPC6 knockout promotes autophagic flux upon IRI. Besides western blot assays, we observed the immunohistochemical staining of CC3 in tissue sections and TECs. The staining intensity of CC3 antibody was found to be notably elevated in WT-I/R tissue sections compared with WT-sham, the expression of which was relatively higher than that in TRPC6^*−/−*^ mice that had been subjected to the same surgery (Figure 4C). In summary, we found that TRPC6 influences IRI and that TRPC6 knockout augments autophagy and mitigates the occurrence of apoptosis.

HIF-1α is a central regulator in the cellular adaptation to hypoxia condition during I/R treatment. We analyzed the levels of HIF-1α and its downstream effector protein AurA in WT and TRPC6^−/−^ mice upon I/R. We found that HIF-1α and AurA were both up-regulated under I/R treatment, while only HIF-1α but not AurA was decreased in TRPC6^−/−^ mice under the same condition (Supplementary Figure S1A). We also analyzed the level of polycystin 2 (PC2), a protein encoded by the PKD2 gene in TECs (Menè et al., 2013). Results showed that there is no remarkable change in the PC2 expression in both WT and TRPC6^−/−^ mice upon I/R (Supplementary Figure S1B).

### TRPC6 Knockout Partly Restores Calcium Influx and Apoptosis Induced by H/R *In Vitro*

**FIGURE 5:**
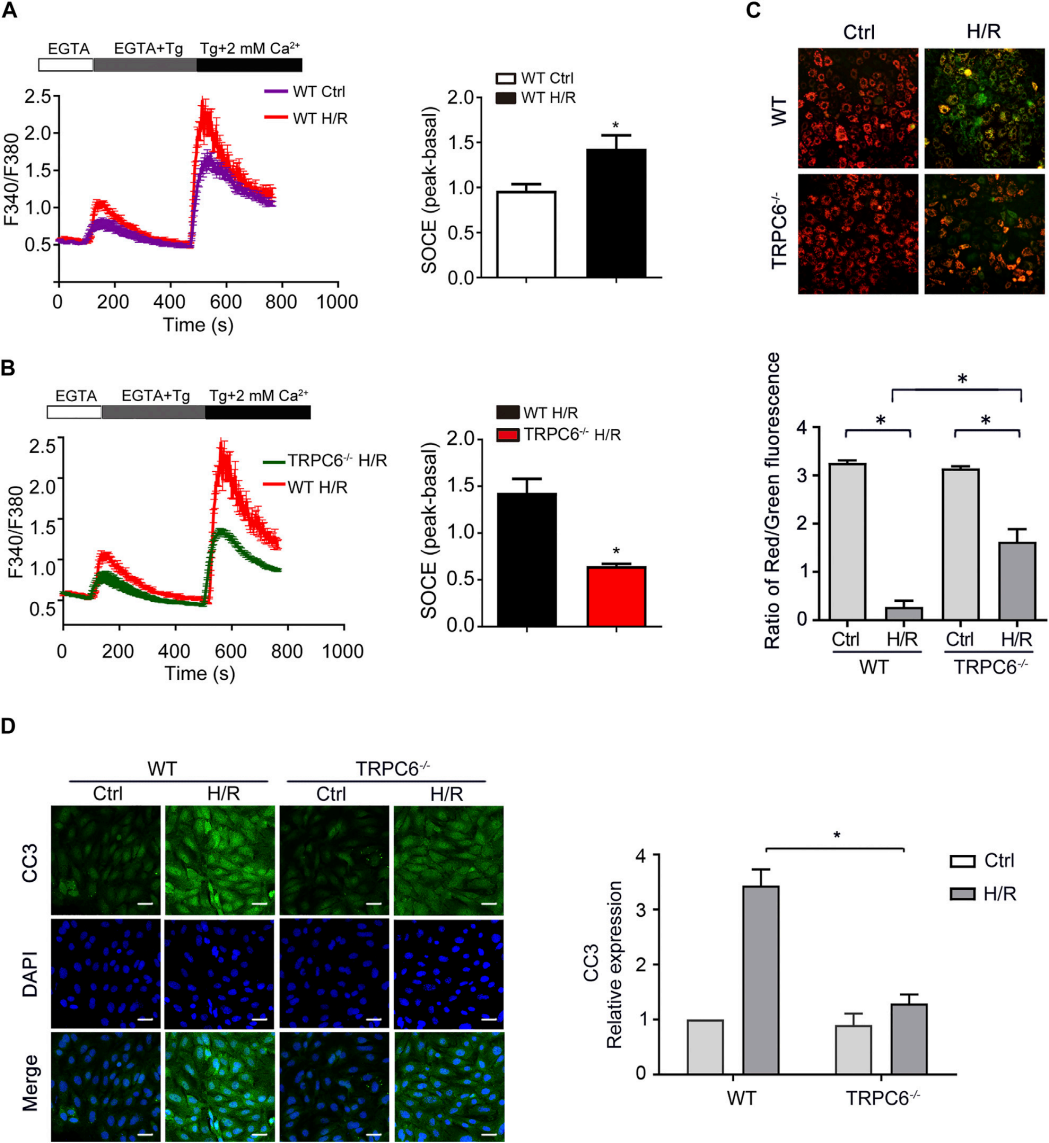
TRPC6 knockout partly restores calcium influx and apoptosis induced by H/R *in vitro*. **(A, B)** Representative traces showing thapsigargin-induced SOCE after H/R treatment in WT and TRPC6^−/−^ mice. Quantification of peak SOCE values is expressed as mean ± SEM, *n* = 3 (40–60 cells per experiment), ^*^ *p* < 0.05. **(C)** MMP changes of primary TECs after H/R injury were measured with JC-1 dye. Fluorescent images were photographed by a fluorescence microscope at a magnification of ×200 (scale bar: 50 μm). Bars represent mean ± SEM, *n* = 10, ^*^ *p* < 0.05. **(D)** Representative confocal microscopy images of CC3 expression in primary TECs from WT and TRPC6^−/−^ mice upon H/R treatment (photographed at a magnification of ×200, scale bar: 50 μm). Bars represent mean ± SEM, *n* = 6, ^*^ *p* < 0.05.

Store-operated Ca^2+^ entry (SOCE), activated by depletion of intracellular Ca^2+^ stores, is the principal means of Ca^2+^ influx in non-excitable cells, including TECs. Thapsigargin (Tg) (a sarcoplasmic reticulum Ca^2+^ ATPase inhibitor) was used to evaluate the function of TRPC6-triggered SOCE in primary TECs. Calcium imaging results showed that H/R treatment increased SOCE (Figure 5A). More importantly, H/R-triggered SOCE was obviously reduced in TRPC6^−/−^ TECs than WT TECs (Figure 5B). Given the data showing that H/R treatment increases TRPC6 expression, this could prove that increased TRPC6 protein expression leads to more functional TRPC6 channels and increased SOCE. Due to ischemic stress, abnormal Ca^2+^ accumulated in the mitochondria causes Ca^2+^ overload leading to the alteration of the mitochondrial membrane permeability transition pore (mPTP), leading to mitochondrial dysfunction and cell death. MMP was assessed by JC-1 dye staining. With the damage of mitochondria, they appeared to immediately depolarize, accompanied by JC-1 discharging from the mitochondria to the cytoplasm and fluorescence shifting from red (JC-1 aggregates) to green (JC-1 monomers). The fluorescent activity of JC-1 presented highly increased green fluorescence in WT TECs after H/R treatment (Figure 5C). MMP, however, was partially reversed in the context of TRPC6^*−/−*^ after TECs followed by H/R injury (Figure 5C), indicating that TRPC6^*−/−*^ ameliorates mitochondrial function and reduces apoptosis. More importantly, TECs that have undergone H/R treatment in WT mice showed that the fluorescent intensity of CC3 was largely augmented than that of untreated cells. TRPC6^*−/−*^ alleviated the amount of CC3 under the same condition (Figure 5D).

### TRPC6 Knockout Reduces TEC Apoptosis by Down-Regulating PI3K/AKT and ERK1/2 Pathways

**FIGURE 6:**
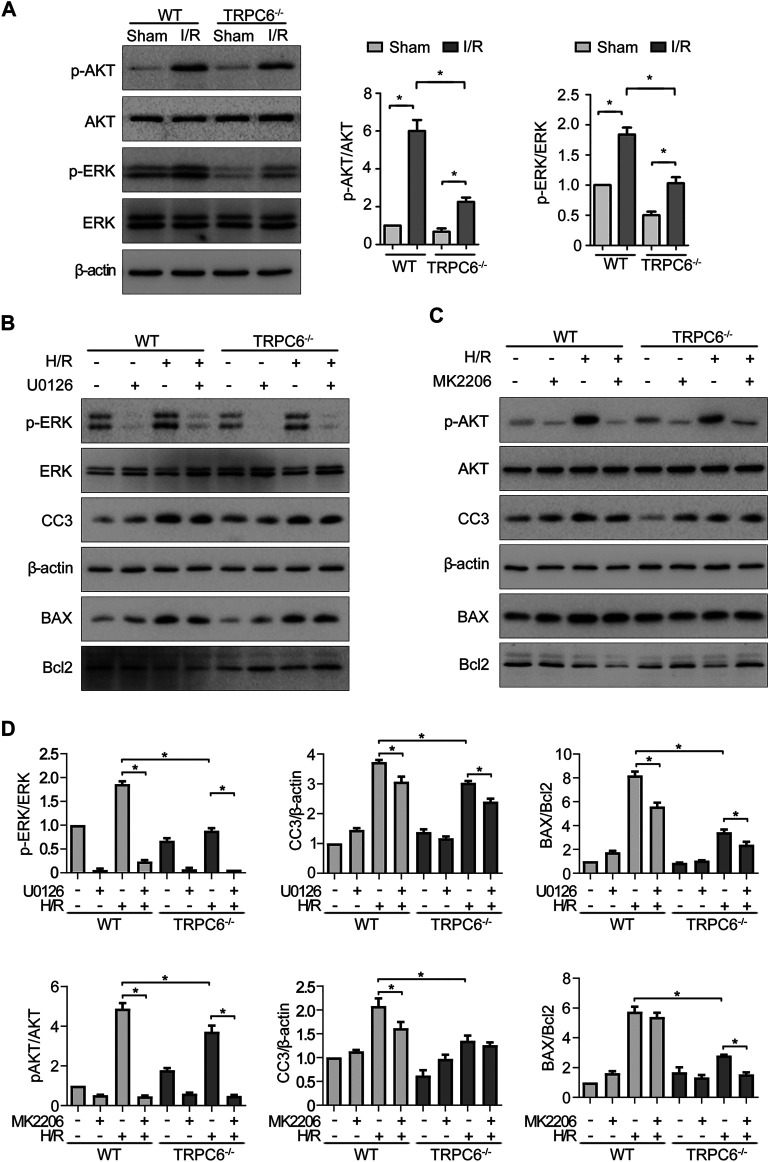
TRPC6 knockout reduces TEC apoptosis by down-regulating PI3K/AKT/mTOR and ERK1/2 pathways. **(A)** Representative western blot images show the level of phosphorylated and total AKT and ERK1/2 expressions of tissue lysates extracted from WT and TRPC6^−/−^ mice. Bars represent mean ± SEM, *n* = 3, ^*^ *p* < 0.05. **(B, C)** Representative western blot images show the level of phosphorylated and total AKT and ERK1/2, BAX, Bcl-2, and CC3 expressions after primary TECs subjected to H/R treatment and addition with inhibitors of AKT (MK2206) and ERK (U0126). **(D)** Bar graphs show the relative quantification of p-AKT/AKT, p-ERK/ERK, CC3/β-actin, and BAX/Bcl-2. Data are expressed as mean ± SEM, *n* = 3, ^*^ *p* < 0.05.

**FIGURE 7:**
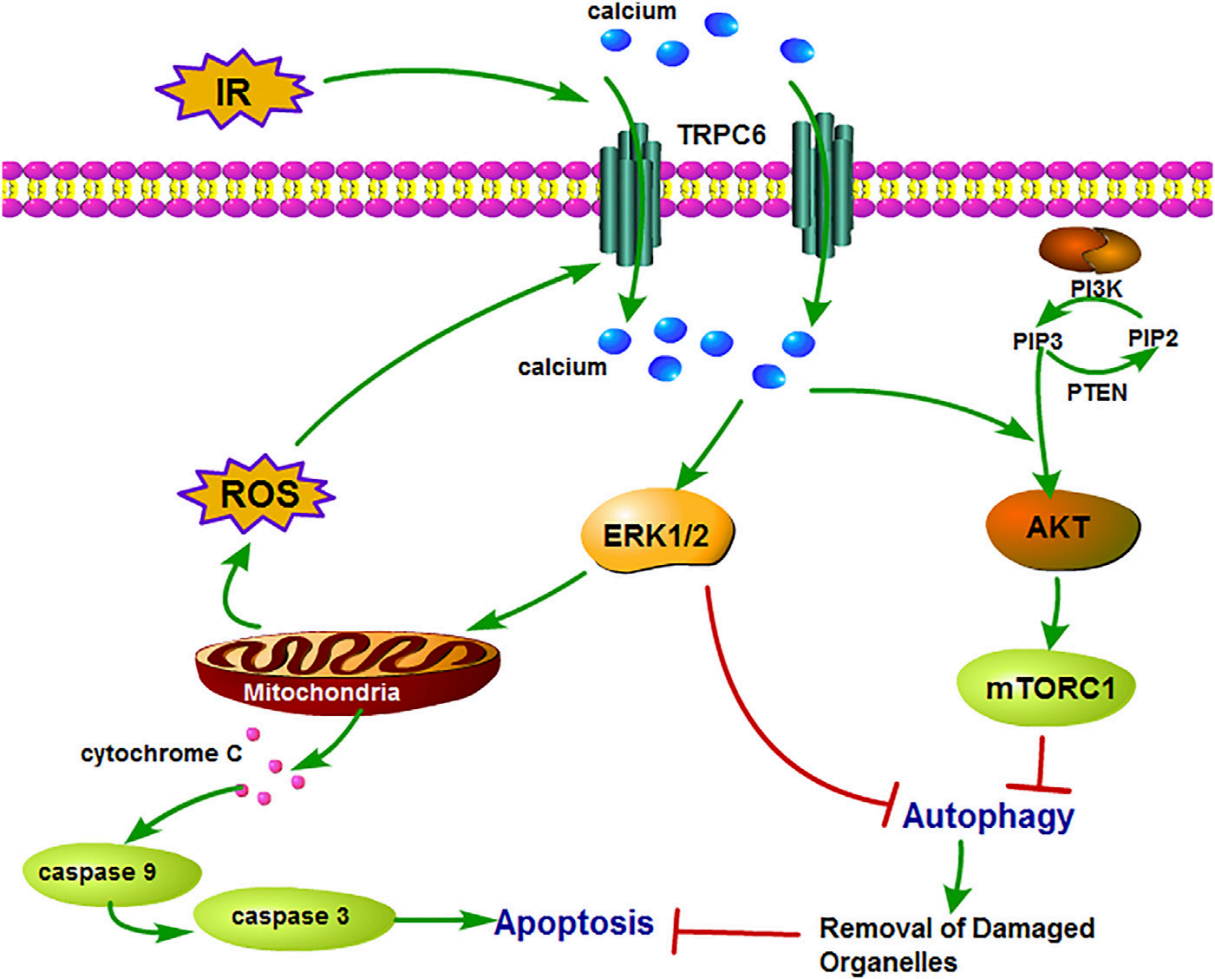
Schematic diagram of mechanism of TRPC6 in renal IRI. TRPC6 is overexpressed during IRI and leads to damaged Ca^2+^ influx into TECs, which activates ERK and PI3K/AKT signaling pathways. Then, these two pathways lead to cell apoptosis by inhibiting cytoprotective autophagy which can remove the damaged organelles upon IRI. Moreover, the activation of the ERK pathway induces excessive ROS production from the mitochondria and results in aggravating apoptosis.

To gain insight into the role of TRPC6 in AKT and ERK1/2 signaling following oxidative stress damage, we evaluated the protein level of p-AKT and p-ERK. As shown in Figure 6A, western blot analysis from the *in vivo* experiment revealed that IRI gave rise to phosphorylation of AKT and ERK in both WT and TRPC6^*−/−*^ mice. On the contrary, the increased levels of p-AKT and p-ERK in TRPC6^*−/−*^ *-*I/R were lower than those in WT counterparts (Figure 6A). Subsequently, we utilized the inhibitors of AKT (MK2206) and ERK (U0126) to reconfirm the signaling pathways involved in apoptosis regulation. In agreement with IRI, both kinases of p-AKT and p-ERK showed the identical variations during H/R treatment. Moreover, after the respective addition of MK2206 and U0126 in H/R primary TECs, it could be observed that the phosphorylation of AKT and ERK was dramatically reduced. The level of BAX/Bcl-2 ratio and CC3 decreased somewhat compared to that in mere H/R treatment without the presence of inhibitors (Figures 6B,C). Furthermore, TRPC6^*−/−*^ exposed fewer CC3 protein expressions and lower BAX/Bcl-2 protein ratio than WT counterparts after addition of the aforementioned inhibitors upon H/R (Figure 6D). Taking all the data into account, TRPC6-Ca^2+^, AKT, and ERK1/2 signaling is responsible for apoptosis regulation. Accordingly, TRPC6 knockout reduces TEC apoptosis by down-regulating PI3K/AKT and ERK1/2 pathways as depicted in Figure 7.

## Discussion

AKI is a serious health problem with urgent need for effective treatment strategies. To our knowledge, our study is the first to implement the *in vivo* I/R-induced AKI model to investigate the role of TRPC6 gene deletion in mice. In the current study, we discovered the following: 1) Renal I/R distinctly gave rise to intracellular Ca^2+^ overload and enhanced the expression of TRPC6. 2) TRPC6 knockout protected the kidney against IRI through enhancing autophagy and diminishing apoptosis. 3) The potential reno-protective mechanism of TRPC6^*−/−*^ was connected with PI3K/AKT and ERK1/2 pathways. The current results indicated that TRPC6^*−/−*^ mice confer a remarkable reno-protective effect against renal IRI, restraining the increase in Scr and BUN, relieving the renal tubular lesion, and decreasing TEC apoptosis compared with WT mice. In addition, the underlying mechanism of the anti-apoptotic effect of TRPC6^*−/−*^ in TECs is connected with negatively regulating PI3K/AKT and ERK1/2 pathways.

Past studies have provided abundant evidence illuminating that Ca^2+^ overload and oxidative stress are main triggers of organ IRI (Legrand et al., 2008; Nieuwenhuijs-Moeke et al., 2020). Recently, more attention has been focused on the TRPC subfamily functioning as widely expressed “cellular sensors” and “cellular effectors” of cell homeostasis (Clapham, 2003; Sakaguchi and Mori, 2020). TRPC6, as a non-selective Ca^2+^-permeable cation channel, is sensitive to the intracellular concentration of Ca^2+^ and plays a critical role in many cellular functions. Daria *et al.* reported that TRPC6 participates in the regulation of podocyte Ca^2+^ and renal damage in diabetic kidney disease (Ilatovskaya et al., 2018). Besides, our recent study showed that TRPC6 regulates the epithelial–mesenchymal transition of TECs and relates to kidney fibrosis (Zhang et al., 2020). Taken together, these studies showed the same results that TRPC6 blockage plays a beneficial role under pathological conditions. In accordance with this, H&E staining, PAS staining, and renal function measurement analysis in our study revealed that TRPC6^*−/−*^ mice were not affected under physiological conditions, while renal tissue damage was mitigated under IRI compared to that in WT mice. These findings suggest that TRPC6 inhibition may be conducive to reducing structural and functional kidney impairment during IRI.

Ca^2+^ entry through TRPC6 channels has been shown to elevate cytoplasmic Ca^2+^. Intracellular Ca^2+^ increased during the reperfusion period. It has been confirmed by our group that H_2_O_2_-induced SOCE was clearly reduced after TRPC6 deletion (Hou et al., 2018). Next, our results showed that TRPC6 expression in tubules increased upon IRI. Aberrant intracellular Ca^2+^ accumulation causes mitochondrial Ca^2+^ overload as a consequence of depolarization of MMP. The major mechanism is large amounts of Ca^2+^ leading to the uncoupling of mitochondrial respiratory chain and overproduction of ROS, which in turn lead to swelling of the mitochondrial membrane, the activation of mPTP, and thus the release of cytochrome c and other pro-apoptotic factors (Seidlmayer et al., 2015). In this study, our results showed distinct induction of apoptosis in renal tissue, and TRPC6^*−/−*^ mice exhibited lower apoptosis compared to WT mice. Taking the above findings into consideration, it is possible that TRPC6-mediated Ca^2+^ signaling further augments ROS activity due to increased production of superoxide, thereby causing a feedforward regulation and amplification of ROS/TRPC6 signaling in I/R-induced TEC injury. On the contrary, TRPC6^*−/−*^ alleviates Ca^2+^ overload, then mitigates kidney IRI, and decreases the apoptosis rate of TECs, supporting the idea that TRPC6 was involved in kidney dysfunction.

Although Ca^2+^ has been commonly accepted as an inducer of autophagy, it remains unclear how Ca^2+^ regulates autophagy. It has been shown previously that oxidative stress triggers TRPM2-mediated Ca^2+^ influx to inhibit the induction of autophagy *via* CAMK2-BECN1 signaling (Wang et al., 2016). In addition, our previous results showed *in vitro* that TRPC6-mediated Ca^2+^ influx could inhibit TEC autophagy. Both the basic autophagy level and the autophagy level after H_2_O_2_ treatment were significantly higher in TRPC6^−/−^ primary TECs than in WT TECs (Hou et al., 2018). Therefore, we speculated that, in renal IRI, TRPC6 may aggravate TEC apoptosis and kidney injury by inhibiting autophagy. To verify the above hypothesis, we used WT and TRPC6^−/−^ mice to prepare the I/R model *in vivo* and detected the change of autophagy-related proteins LC3-II and p62 to explore the regulation of TRPC6 on TEC autophagy upon I/R. Our results show that TRPC6 knockout enhances I/R-induced autophagy. Thus, the overexpression of TRPC6 upon I/R causes excessive Ca^2+^ influx and mitigates cytoprotective autophagy of TECs so as to increase apoptosis upon I/R treatment.

This study indicates an inseparable association between TRPC6 and TEC apoptosis in renal injury, which was confirmed by observing that pro-apoptotic markers (CC3 and BAX) were obviously increased by H/R treatment, and this phenomenon was decreased in TRPC6^*−/−*^ cells. Our data support that the removal of TRPC6 attenuates TEC apoptosis by decreasing the phosphorylation of AKT. We found that p-AKT was significantly increased after H/R in WT compared with TRPC6^*−/−*^ and that the AKT inhibitor with MK2206 further ameliorated the anti-apoptotic effect of TRPC6 deletion. Moreover, reports suggest that ERK1/2 activation was linked to renal injury and apoptosis instead of contributing to cell survival (Zhuang and Schnellmann, 2006; Alderliesten et al., 2007). The scholars believed that ERK1/2 regulates upstream factors in apoptotic events through downstream transcription factors, inducing the release of cytochrome c, down-regulating the anti-apoptotic protein Bcl-2, and up-regulating the pro-apoptotic protein BAX *via* activating and up-regulating the expression of caspases 3, 8, and 9, thereby inducing apoptosis (Lavoie et al., 2020). The results of our study showed that I/R activates ERK1/2 and increases the amount of p-ERK1/2. The apoptotic rate was decreased by means of blocking ERK1/2 using U0126, and the increased level of p-ERK1/2 in TRPC6^*−/−*^ was partially reversed. Based on these data, TRPC6 is more likely to increase apoptosis *via* activation of AKT and ERK1/2 pathways.

In summary, our study reveals that TRPC6 knockout protects TECs against IRI by reducing activation of AKT and ERK1/2, increasing the level of autophagy, and restraining mitochondria-mediated apoptosis. Our study contributes to a comprehensive understanding of the biological function of TRPC6 as well as to the analysis of the pathological mechanism of TEC damage in renal I/R. TRPC6-directed intervention may provide treatment ideas for I/R-induced AKI in the future.

## Data Availability

The original contributions presented in the study are included in the article/Supplementary Material, and further inquiries can be directed to the corresponding authors.
