# Supplementary material for: The Role of TRPC6 in Renal Ischemia/Reperfusion and Cellular Hypoxia/Reoxygenation Injuries
Source: Front Mol Biosci. 2021 Jul 8;8:698975. doi: 10.3389/fmolb.2021.698975 (PMC8295989; doi:10.3389/fmolb.2021.698975)

Supplementary Figure

Supplementary Figure 1. TRPC6 knockout partly restores the increase of HIF-1α induced by I/R treatment, but has no significant effect on aurora kinase A (AurA) and polycystin 2 (PC2) protein expression. **(A)** Representative western blot images of HIF-1 α and AurA expression in renal tissue lysates extracted from WT and $TRPC6^{-/-}$ mice subjected to I/R operations. Data are expressed as means ± SEM, n=3, $^{*}P < 0.05$, NS indicates not significant. **(B)** Representative western blot images of PC2 expression in renal tissue lysates extracted from WT and $TRPC6^{-/-}$ mice subjected to I/R operations. Data are expressed as means ± SEM, n=3, NS indicates not significant.

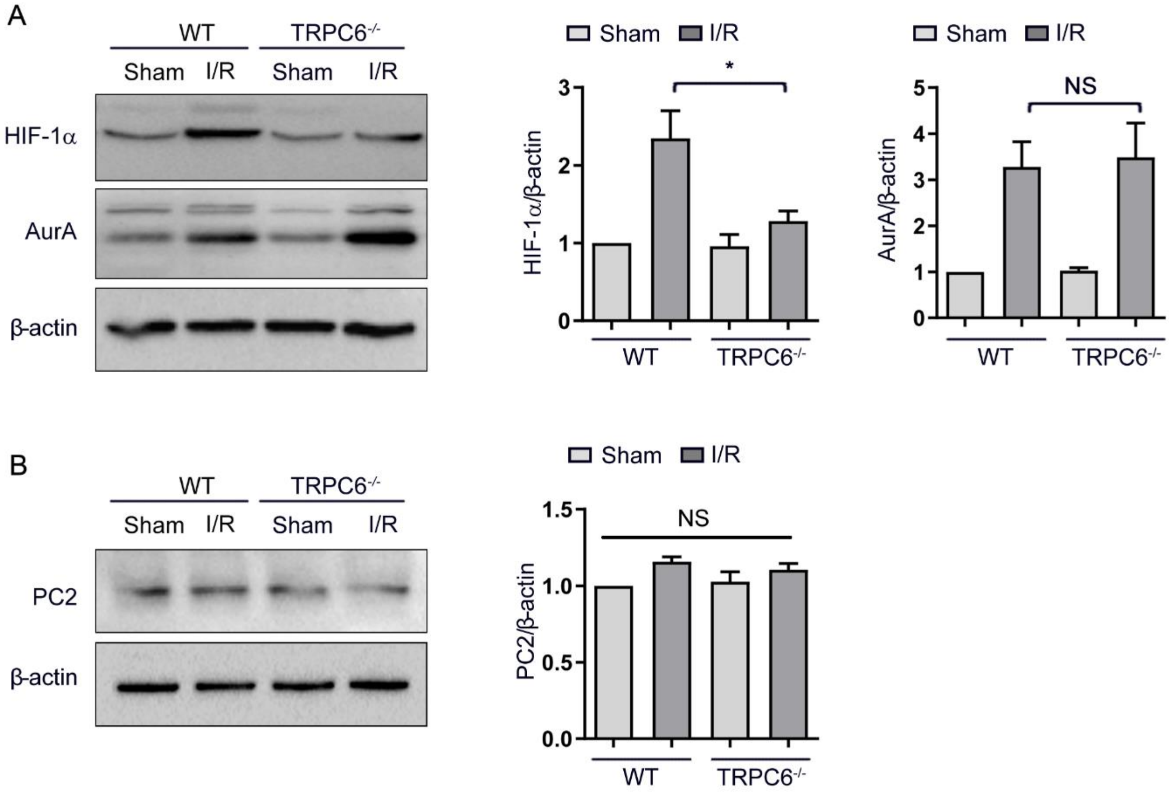

Supplement: Supplementary file 1 [file DataSheet1.pdf]
